# Antibody Fc Glycosylation Discriminates Between Latent and Active Tuberculosis

**DOI:** 10.1093/infdis/jiz643

**Published:** 2020-02-15

**Authors:** Lenette L Lu, Jishnu Das, Patricia S Grace, Sarah M Fortune, Blanca I Restrepo, Galit Alter

**Affiliations:** 1 University of Texas Southwestern Medical Center, Dallas, Texas, USA; 2 Ragon Institute of Massachusetts General Hospital, Massachusetts Institute of Technology and Harvard, Cambridge, Massachusetts, USA; 3 Department of Immunology and Infectious Diseases, Harvard T.H. Chan School of Public Health, Boston, Massachusetts, USA; 4 School of Public Health, University of Texas Health Houston, Brownsville, Texas, USA; 5 South Texas Diabetes and Obesity Institute, University of Texas Rio Grande Valley, Edinburg, Texas, USA

**Keywords:** tuberculosis, antibodies, glycosylation, Fc effector function, diagnostics

## Abstract

**Background:**

*Mycobacterium tuberculosis* remains a global health problem and clinical management is complicated by difficulty in discriminating between latent infection and active disease. While *M. tuberculosis*-reactive antibody levels are heterogeneous, studies suggest that levels of IgG glycosylation differ between disease states. Here we extend this observation across antibody domains and *M. tuberculosis* specificities to define changes with the greatest resolving power.

**Methods:**

Capillary electrophoretic glycan analysis was performed on bulk non-antigen–specific IgG, bulk Fc domain, bulk Fab domain, and purified protein derivative (PPD)- and Ag85A-specific IgG from subjects with latent (n = 10) and active (n = 20) tuberculosis. PPD-specific isotype/subclass, PPD-specific antibody-dependent phagocytosis, cellular cytotoxicity, and natural killer cell activation were assessed. Discriminatory potentials of antibody features were evaluated individually and by multivariate analysis.

**Results:**

Parallel profiling of whole, Fc, and Fab domain-specific IgG glycosylation pointed to enhanced differential glycosylation on the Fc domain. Differential glycosylation was observed across antigen-specific antibody populations. Multivariate modeling highlighted Fc domain glycan species as the top discriminatory features, with combined PPD IgG titers and Fc domain glycans providing the highest classification accuracy.

**Conclusions:**

Differential glycosylation occurs preferentially on the Fc domain, providing significant discriminatory power between different states of *M. tuberculosis* infection and disease.

## Teaser

Clinical management of *Mycobacterium tuberculosis* is complicated by difficulty in discriminating between latent infection and active disease. Here profiling antibody features and multivariate modeling highlight Fc domain glycosylation as a top discriminatory feature.

Tuberculosis is the leading cause of infectious disease deaths worldwide [1]. Current diagnostic tools are suboptimal with nonspecific clinical symptoms and poorly sensitive and resource-demanding microbial-based tests such as acid-fast smears and culture or nucleic acid amplification. Moreover, T-cell based tuberculin skin tests and interferon-γ (INF-γ) release assays do not distinguish between individuals with latent infection who are clinically well and the 5%–10% of this population who progress to active tuberculosis disease, the primary source of transmission and mortality if untreated [2]. Thus, a simple point-of-care diagnostic with an enhanced ability to distinguish latent from active tuberculosis could dramatically limit disease [3].

Recent studies demonstrate that host immune responses as measured by blood inflammatory transcripts [4] and immune complex activity [5] correlate with latent and active tuberculosis [6]. However, because these measures are not microbe specific, utility as a biomarker for infection is uncertain. While antigen-specific T-cell mediated immunity is critical in preventing the acquisition and progression of disease [7], current antigen-specific T-cell–based tests fail to distinguish latent from active tuberculosis [2]. Conversely, while the role of humoral immunity in the control of *Mycobacterium tuberculosis* is less clear and the overall magnitude of the antibody (Ab) response represents an unreliable biomarker [8], Ab constant (Fc) domain features shift with disease states, resolving latent and active tuberculosis [5].

Shifts in Ab glycosylation have been observed with autoimmune disease [9, 10], age [11], pregnancy [12], and HIV infection [13, 14]. Data from mice and also humans from 2 distinct geographic regions have noted Ab changes with tuberculosis disease severity [5, 15, 16]. Specifically, Abs in patients with active tuberculosis are agalactosylated, similar to those with autoimmune flares [16]. Importantly, these changes were observed across total circulating and antigen-specific IgG.

*N*-linked glycosylation on a single conserved site on the CH2 domain of the IgG Fc is critical for downstream effector functions [17]. This glycan structure consists of a core biantennary complex of *N*-acetyl glucosamine and mannose residues. This core is further extended with sialic acid (SA), galactose (G), fucose (F), and a bisecting *N*-acetyl glucosamine (GlcNAc) [17], which in combination generate up to 36 unique glycoforms that may be attached to the Fc domain [17]. These changes modify Fc affinity for Fc receptors on immune cells, regulating a variety of cellular effector functions including cytotoxicity, phagocytosis, antigen presentation, and inflammation [18]. Moreover, IgG glycosylation has been noted to diverge across antigen specificities within a given individual [13, 19–23], suggesting that Ab glycosylation may develop in an antigen-specific manner.

Beyond Fc glycosylation, approximately 20% of IgG is glycosylated on the antigen binding (Fab) domain, due to stochastic incorporation of *N*-glycan sites during somatic hypermutation [24, 25]. Thus, the Fab represents an additional source of glycan heterogeneity detected from the whole Ab. Whether *M. tuberculosis* infection-associated glycan changes occur across the whole Ab, or preferentially on the Fc or Fab domain, or on particular antigen-specific Ab subpopulations is unclear, but could provide further insights into biomarkers of tuberculosis disease state.

The aim of this study was to evaluate the potential for differential Ab profiles to discriminate between latent and active tuberculosis. To begin to define the specific Ab glycan changes, glycosylation profiles were measured on whole, Fc, and Fab fragments of circulating and *M. tuberculosis* purified protein derivative (PPD)- or Ag85A-specific IgG [26]. Alterations in bulk IgG glycosylation between latent and active tuberculosis largely correlated with Fc domain changes. Analysis incorporating all Ab features revealed that Fc domain glycosylation provided the greatest resolution of disease states, particularly when combined with measures of PPD Ab titers.

## METHODS

### Study Population

Adult HIV-seronegative subjects with latent (n = 10) and active (n = 20) tuberculosis were recruited in south Texas (Hidalgo County Department of Health and Human Services) and Mexico (Secretaria de Saluld de Tamaulipas). A diagnosis of active tuberculosis was based on *M. tuberculosis* isolation from sputum and latent tuberculosis on a positive Quantiferon-Gold (Qiagen) or T.Spot.TB (Oxford Immunotec) assay. Data on age, gender, BCG vaccination status, glycated hemoglobin (HbA1C), and body mass index (BMI) were collected [27] (Supplementary Table). All study participants gave written, informed consent. The study was approved by the institutional review boards of the participating institutions.

### Isolation of Whole Bulk IgG

Total IgG was purified from plasma using Melon Gel resin (ThermoScientific) and filtered through 0.2-µM (Fisher) and 300-kDa filters (Amicon).

### Isolation of Fc and Fab Domains

To cleave whole IgG into Fc and Fab domains, whole IgG (20 µg) was digested with IdeS (Promega) at 37°C for 1 hour. Fc domains were isolated with protein G beads (Millipore) at room temperature for 1 hour. The supernatant containing the Fab fragments was removed for further processing. Glycans were isolated from samples containing whole IgG, Fab, or Fc fragments and labeled using Glycan Assure APTS kit (Life Technologies, A28676).

### Isolation of PPD- and Ag85A-specific IgG Glycans

PPD (Statens Serum Institute) or recombinant Ag85A (BEI) were biotinylated with sulfosuccinimidyl-6-[biotinamido]-6-hexanamido hexanoate (sulfo-NHS-LC-LC biotin; ThermoScientific) and coupled to streptavidin columns (Agilent) through which plasma was passed, then washed with phosphate-buffered saline (PBS; Corning). Antigen-specific Abs were eluted using 100 mM citric acid (pH 3.0) and neutralized with 0.5 M potassium phosphate (pH 9.0). IgG was purified from the eluted antigen-specific Abs by protein G beads. *N*-linked glycans from IgG samples were cleaved by peptide-*N*-glycosidase F (NEB) [28]. Proteins were precipitated in ice-cold ethanol. Glycan-containing supernatants were dried by CentriVap, labeled with a 1:1 ratio of 50 mM 8-aminoinopyrene-1,3,6-trisulfonic acid (APTS; ThermoFisher) in 1.2 M citric acid and 1 M sodium cyanoborohydride in tetrahydrofuran (Sigma-Aldrich) at 55°C for 2 hours with unbound APTS removed using Bio-Gel P-2 (Bio-Rad) size exclusion resin.

### Analysis of Glycans

APTS-labeled samples were run with a LIZ 600 DNA ladder in Hi-Di formamide (ThermoFisher) on an Applied Biosystems 3500/3500xL Genetic Analyzer and analyzed with GlycanAssure Data Acquisition Software v.1.0.

### PPD-specific IgG Quantitation

To determine PPD-specific IgG titers, enzyme-linked immunosorbent assay (ELISA) plates (Nunc) were coated with PPD (250 ng/mL) or PBS-5% bovine serum albumin (BSA) at 4°C for 16 hours and blocked with PBS-5% BSA at room temperature for 2 hours. IgG samples were incubated at room temperature for 2 hours. HRP-conjugated anti-human IgG (1:500 in PBS; R&D Systems) was incubated at room temperature for 1 hour. Wells were developed in 0.4 mg/mL *o*-phenylenediamine in PBS/H_2_O_2_ and stopped by 2.5 M H_2_SO_4_. Absorbances were measured at 450 and 570 nm.

### Antigen-Specific IgG Subclass Quantitation

Relative levels of antigen-specific Ab subclasses were quantified by customized Luminex [29]. Carboxylated microspheres (Luminex) were coupled with PPD, Ag85A, *M. tuberculosis* culture filtrate (BEI), and *M. tuberculosis* soluble protein (BEI) by covalent *N*-hydroxysuccinimide (NHS)-ester linkages via 1-ethyl-3-(3-dimethylaminopropyl)carbodiimide hydrochloride (EDC) and NHS (ThermoScientific). Antigen-coated microspheres (5000/well) were added to each sample (5 µg bulk IgG) in 5 replicate wells of a 96-well plate (Millipore) and incubated at 4°C for 16 hours. Microspheres were washed, and IgG1-, IgG2-, IgG3-, IgG4-, or bulk IgG-specific detection reagents (Southern Biotech) were added at room temperature for 2 hours. Beads were read on a Bio-Plex 200 System. The background signal (mean fluorescence intensity [MFI] of microspheres incubated with PBS) was subtracted.

### THP1 Phagocytosis Assay

PPD was biotinylated with sulfo-NHS-LC BIOTIN (ThermoFisher) and incubated with 1 μm fluorescent neutravidin beads (Invitrogen) at 4°C for 16 hours. Excess antigen was washed away. Antigen-coated beads were incubated with IgG samples (100 µg/mL) at 37°C for 2 hours to which THP1 cells (1 × 10^5^/well) were added and incubated further at 37°C for 16 hours. Bead uptake in fixed samples was measured on a BD LSRII. The integrated MFI (% bead-positive frequency × MFI/10 000) generated phagocytic scores [30].

### Antibody-Dependent Cellular Cytotoxicity Assay

A modified rapid fluorometric antibody-dependent cellular cytotoxicity assay was used [31, 32]. CEM-NKr CCR5^+^ cells (National Institutes of Health AIDS Reagent Program) were pulsed with PPD (60 µg/mL) at room temperature for 1 hour and labeled with the intracellular dye 5(6)-carboxyfluorescein diacetate *N*-succinimidyl ester

(CFSE; Sigma) and membrane dye PKH26 (Invitrogen). NK cells were isolated from seronegative donor whole blood with RosetteSep (Stem Cell Technologies). Purified IgG (100 µg/mL) was added to the labeled CEM-NKr cells (2 × 10^4^/well) and incubated with NK cells (2 × 10^5^/per well) at 37°C for 4 hours. The proportion of PKH26^+^ cells lacking intracellular CFSE staining (% dead cells) in fixed samples was determined by flow cytometry.

### Ab-Dependent NK Cell Activation

ELISA plates were coated with PPD (300 ng/well) or BSA at 4°C for 16 hours [33]. Purified IgG (25 µg) from study participants was added to each well. NK cells were isolated from whole blood from seronegative donors with RosetteSep. NK cells (5 × 10^4^/well) were incubated with anti-CD107a–phycoerythrin (PE)–Cy5 (BD), brefeldin A (10 mg/mL) (Sigma), and GolgiStop (BD) at 37°C for 5 hours. Cells were stained for surface markers using anti-CD16–allophycocyanin (APC)–Cy7 (BD), anti-CD56–PE–Cy7 (BD), and anti-CD3–AlexaFluor 700 (BD), and then intracellularly with anti-IFN-γ–APC (BD) and anti-MIP1β–PE (BD) using Fix and Perm A and B solutions (ThermoFisher). Frequency (%) of NK cells positive for CD107a, IFN-γ, and MIP1β were determined with NK cells defined as CD3^−^ and CD16/56^+^.

### Ab-Dependent Neutrophil Activation

Healthy donor whole blood was mixed with equal volume of 3% dextran-500 (ThermoFisher) at room temperature for 25 minutes to pellet red blood cells. Leukocytes were removed, washed in Hank's Balanced Salt Solution (HBSS) without calcium and magnesium (ThermoFisher), and separated using Ficoll-Histopaque (Sigma-Aldrich) centrifugation. The granulocyte pellet was harvested. PPD-coated beads were incubated with IgG (100 µg/mL) at 37°C for 2 hours to which isolated neutrophils (1 × 10^5^/well) were added and incubated further at 37°C for 16 hours. Bead uptake by neutrophils identified by CD66b (BioLegend) was measured by flow cytometry.

### Analysis

Statistical analysis and graphing were performed using GraphPad Prism 7.0, JMP Pro 12, and Matlab in a nonparametric approach to avoid assumptions about data normality. To compare percent Ab sugar levels across whole IgG, Fc, and Fab domains and bulk, PPD- specific, and Ag85A-specific Abs within the same individual, a Friedman statistic value was obtained first. If the Friedman statistic value was <0.05, Wilcoxon matched pairs signed rank was used to evaluate the statistical significance between each combination of 2 groups within the 3. Mann-Whitney tests were used to compare percent glycan levels between different individuals with latent or active tuberculosis. Principal component analysis was performed using default parameters in JMP Pro 12.

While Fab and Fc glycosylation is thought to occur independently [24, 25], it is plausible that relationships may exist among particular features in polyclonal responses. Thus, we applied a multivariate approach to define the minimal set of Ab features that could separate latent and active tuberculosis. Specifically, this modeling utilizes prefeature downselection by the least absolute shrinkage and selection operator (LASSO) [34] and visualization by partial least square discriminant analysis (PLSDA) to ensure that as few features as possible are selected to capture the greatest variance across disease states while avoiding overfitting. Ab effector functions, whole, Fab, Fc, PPD-specific and Ag85A-specific glycans, and IgG titers were features with 4 potential covariates: age, gender, HbA1c, and BMI. Latent and active tuberculosis were binary outputs. There were 30 subjects, each with 193 measurements. Each feature was centered and scaled (Z scores). Robustness was evaluated using 5-fold cross-validation replicates with subjects randomly divided into 5 subsets and used exactly once in the test fold against the 4 subsets that served as the training set. A second internal 5-fold cross-validation using only the training dataset determined the coefficient for the LASSO penalty term. A fold-specific support vector machine [35] classifier (linear kernel with default Matlab parameters) was trained using LASSO-selected features to predict clinical outcomes. Twenty independent 5-fold cross-validation replicates were performed, with visualizations of latent variables by PLSDA on the LASSO-selected features [36]. The identified correlates showed the same trend even without data imputation.

Model performances were measured using 2 independent “irrelevant datasets” with repetitions of 5-fold cross-validation generating a distribution of model classification accuracies: permuted data [37] and randomly selected size-matched features. These irrelevant datasets generated models to establish a baseline against which true model performance was compared. For each control model, processes were repeated 100 times to generate a distribution of accuracies. Predicted clinical outcomes were compared to true clinical outcomes to obtain classification accuracy. Exact *P* values are tail probabilities of true in the distribution of control model classification accuracies. Median *P* values across independent cross-validation replicates are reported.

## RESULTS

### Divergent Glycans on Fc and Fab Domains and Whole IgG

Previous studies pointed to differences in Ab glycosylation profiles between latent and active tuberculosis [5]. However, it was unclear whether these glycan changes reflected altered Fc or Fab glycosylation. Thus we sought to probe Ab domain-specific glycosylation in tuberculosis.

**Figure 1.**
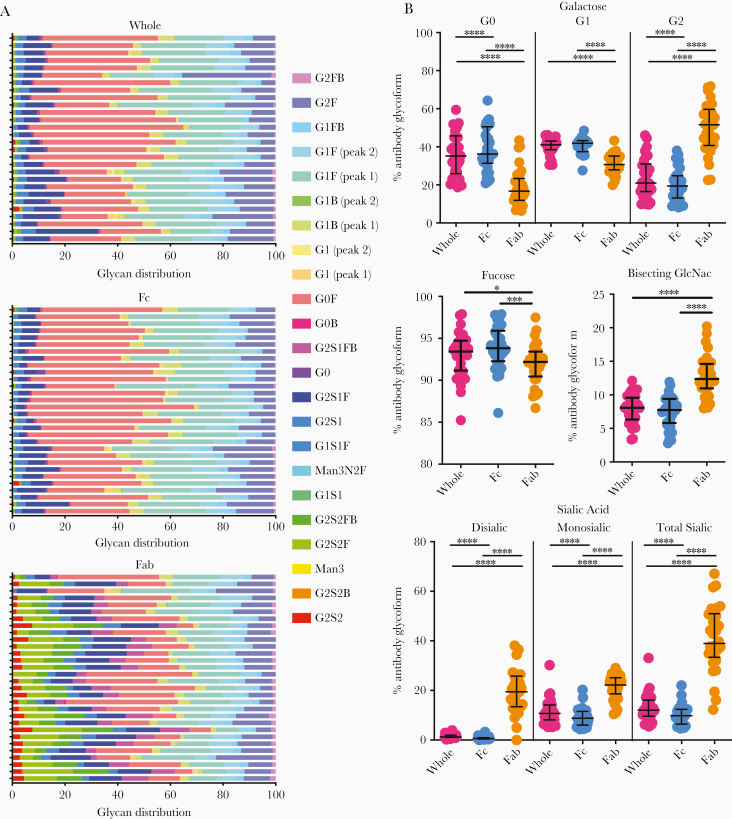
Differential glycosylation of Fc, Fab, and whole antibody (Ab) in tuberculosis. *A*, Heatmap of individual glycan substructures (columns) for each individual (rows) shows differential modifications across whole (top), Fc (middle), and Fab domains (bottom) of IgG. *B*, Modifications of all glycan structures containing galactose (G0, agalactosylated; G1, monogalactosylated; G2, digalactosylated), fucose, bisecting *N*-acetyl glucosamine (GlcNAc), and sialic acid are graphed as percent of total IgG sugar modifications, separated by region (whole Ab, Fc only, and Fab only). Each dot represents an individual with latent (n = 10) or active (n = 20) tuberculosis. Bars represent median and interquartile range. Statistical significance was determined by Friedman statistic to evaluate for differences between 3 paired groups and, if appropriate, followed by Wilcoxon matched pairs signed rank to evaluate the statistical significance between each paired group. * *P* ≤ .05, ** *P* ≤ .01, *** *P* ≤ .001, **** *P* ≤ .001.

**Figure 2.**
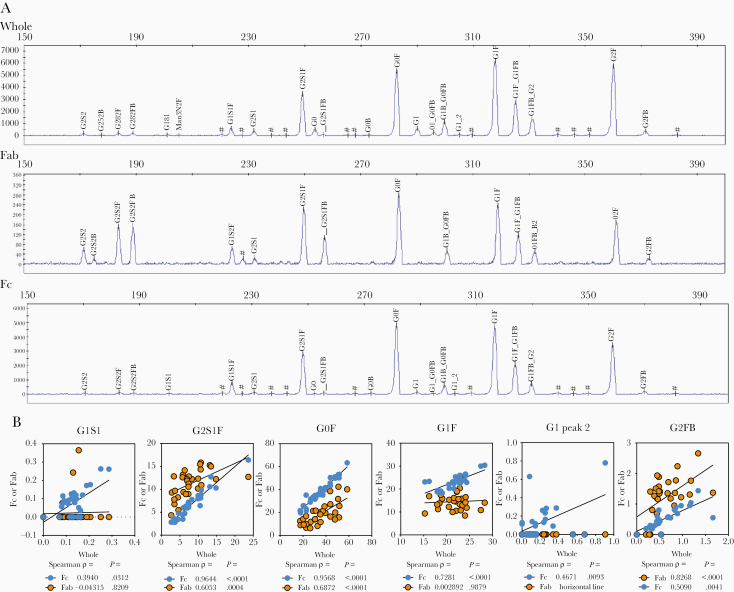
Associations between Fc, Fab, and whole IgG glycans in tuberculosis. *A*, Representative plots generated via capillary electrophoresis of samples from individuals with latent (n = 10) or active (n = 20) tuberculosis are shown for glycans isolated from whole, Fab domain, and Fc domain IgG. Peaks are identified by alignment with standards and relative abundance is calculated by peak areas via GlycanAssure Data Acquisition Software v.1.0. *B*, Prominent peaks from the Fc and Fab domain were correlated by Spearman rank order to whole IgG. Rho (ρ) and *P* values are indicated. Abbreviations: G0, agalactosylated; G1, monogalactosylated; G2, digalactosylated.

A high-throughput capillary electrophoretic approach [28] was employed to analyze released glycans from whole Abs and isolated Fc and Fab domains from plasma-derived IgG of individuals with latent and active tuberculosis. Significant differences in galactose, fucose, bisecting-GlcNAc, and sialic acid were observed across whole, Fc, and Fab domains (Figure 1, Figure 2A, and Supplementary Figure 1) [24]. Consistent with previous reports [12], Fab domains possessed higher levels of digalactosylated (G2), bisected, and sialylated glycan moieties compared to Fc domains (Figure 1B). These differences were most observed in G2S2F, G2S2FB, and G2S1FB (Supplementary Figure 1). Lower relative levels of G0F were detected on the Fab domain compared to the whole and Fc domain (Supplementary Figure 1). For all glycan modifications, Fc glycans correlated more with whole glycan profiles compared to Fab glycan profiles (Figure 2B and Supplementary Figure 1B). These data highlight the dominant contribution of the Fc in driving changes in blood-derived Ab glycan profiles.

### Distinct Glycan Profiles Across Antigen Specificities

**Figure 3.**
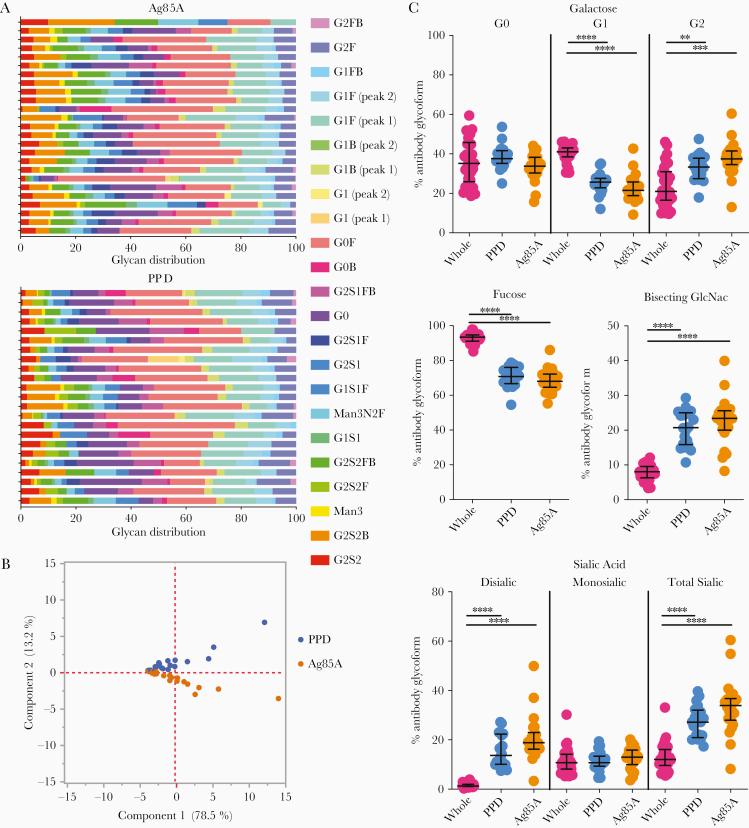
Differential glycosylation of purified protein derivative (PPD), Ag85A, and bulk non-antigen–specific antibody in tuberculosis. *A,* Heatmap shows modifications of glycan substructures (columns) for each individual (rows) for Ag85A (top) and PPD (bottom) specific IgG. *B*, Principal component analysis using all PPD (blue) and Ag85A (orange) glycan data demonstrates the nonoverlapping profiles. *C*, Modifications of all glycan structures containing galactose (G0, agalactosylated; G1, monogalactosylated; G2, digalactosylated), fucose, bisecting *N*-acetyl glucosamine (GlcNAc), and sialic acid are graphed as percent of total IgG sugar modifications, separated by region (whole Ab, PPD only, and Ag85A only). Each dot represents an individual with latent (n = 10) or active (n = 20) tuberculosis. Bars represent median and interquartile range. Statistical significance was determined by Friedman statistic to evaluate for differences between 3 paired groups and, if appropriate, followed by Wilcoxon matched pairs signed rank to evaluate the statistical significance between each paired group. * *P* ≤ .05, ** *P* ≤ .01, *** *P* ≤ .001, ****P ≤ .001.

Beyond the Fab and Fc domains, accumulating data suggest that Ab glycosylation varies across antigen specificities in infections and following vaccination [13, 19–22]. To examine whether distinct Ab glycan changes occurred among different *M. tuberculosis* reactive Ab populations, glycosylation profiles were generated from PPD- [38] and Ag85A-[26] specific Abs, the latter a component of PPD [38] and with T-cell and Ab responses in the context of infection [39, 40] and vaccination [41]. Significant differences were observed between antigen-specific and bulk Ab glycan profiles (Figure 3). Each antigen-specific Ab population was associated with a divergent overall glycan profile (Figure 3B), with PPD- and Ag85A-specific Ab glycosylation more correlated with each other as compared to whole non-antigen–specific Ab glycan populations (Supplementary Figure 2). Similar to our previous observations, antigen-specific Abs had higher levels of digalactosylated, bisected, and disialylated structures [19–21] and lower levels of fucosylated moieties (Figure 3C) [20, 22]. Relative levels of G2S1F, G0F, G1F, G1FB, and G2F were lower while G2S2B, G2S2FB, G2S1, G2S1FB, and G0 were higher in Ag85A- and PPD-specific Ab subpopulations compared to non-antigen–specific Abs (Supplementary Figure 2A). While Ag85A and PPD IgG were more similar to each other compared to total IgG, there were differences between the 2 antigens, most notably in relative levels of G2S1F and G2S1FB (Supplementary Figure 2A). Thus, glycan profiles of antigen-specific Ab subpopulations diverged from bulk Abs and from each other (Figure 3B), highlighting potential distinct regulation of glycosylation across *M. tuberculosis-*specific Abs.

### Diverging Ab Features Across Tuberculosis Disease States

**Figure 4.**
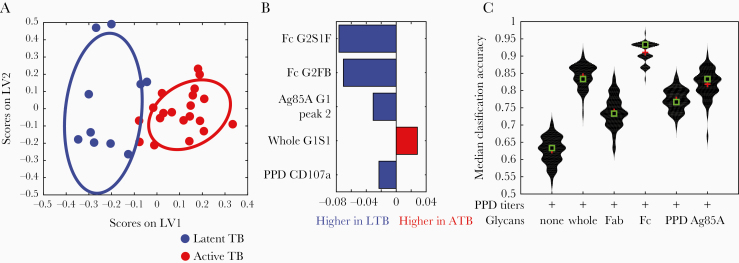
Separation of latent and active tuberculosis by antibody features. *A*, LASSO and PLAS supervised model using all humoral features measured (Ab glycosylation, PPD-specific Fc effector functions, and PPD-specific subclass levels) and demographic data (age and body mass index) identified 5 features that could separate latent (blue dots) and active (red dots) tuberculosis with plot depicting separation. *B*, All 5 features are depicted as VIP scores. *C*, Violin distribution plots show median classification accuracies of PPD-specific Ab levels alone or in combination with whole, Fc, Fab, PPD, or Ag85A IgG glycans. Comparisons between specific groups were used to calculate statistical differences. Abbreviations: Ab, antibody; ATB, active tuberculosis; LASSO, least absolute shrinkage and selection operator; LTB, latent tuberculosis; LV, latent variable; PLAS, partial least square discriminant analysis; PPD, purified protein derivative; TB, tuberculosis; VIP, variable importance in the projection.

To objectively define the specific Ab features corresponding to disease states, Ab glycosylation data were integrated with Ab function, isotype/subclass levels, and demographic data (Supplementary Table). Using purified IgG from individuals with latent or active tuberculosis, PPD-specific Ab-dependent cellular phagocytosis, neutrophil phagocytosis, cellular cytotoxicity, NK cell activation, and IgG1, 2, 3, and 4 levels to PPD, *M. tuberculosis* culture filtrate, and *M. tuberculosis* soluble protein were analyzed (Supplementary Table). A supervised multivariate model incorporating 193 features, including demographic data as potential covariates that could influence Ab function and glycosylation, was utilized to determine the minimal set that could distinguish individuals with latent from active tuberculosis. Feature downselection was performed using a LASSO, then classification using the LASSO-selected features, and visualization using PLSDA [36]. Our model discriminated between latent and active tuberculosis with a median classification accuracy = 0.9 (Figure 4A). Of the 193 features, 5 were sufficient to achieve separation (Figure 4B). Strikingly, 4 of the 5 features were Ab glycan structures: the top 2 features were Fc domain G2S1F and G2FB, followed by Ag85A-specific galactosylation, and the fifth feature was the surface expression of CD107a, a marker of PPD-specific NK cell degranulation (Supplementary Figures 3–7). No demographic feature was identified by the model such that these factors were less likely to represent confounding factors. These analyses highlight the dominant discriminatory power of Ab Fc glycosylation in resolving latent and active tuberculosis.

### Combination of Ag-Specific Ab Titers and Fc Glycans to Discriminate Latent and Active Tuberculosis

While Ab glycan changes have diagnostic potential [9, 10, 13, 14], *M. tuberculosis*-specific Ab titers alone have failed to discriminate between latent and active tuberculosis [8]. However, results suggest that Fc glycans have significant discriminatory power (Figure 4B). To compare these 2 approaches in discriminating latent and active tuberculosis, we used LASSO to predict group classification, and generated median classification accuracies for models using combinations of titer and glycan data (Figure 4C). As expected, PPD-specific titers alone provided poor resolution across groups. In contrast, the addition of glycan data significantly increased classification accuracy, with the best combination resulting in a median classification accuracy of >90% using Fc domain glycans and PPD titers (Figure 4C). Thus, *M. tuberculosis*-specific Ab titers and Fc glycosylation together may enhance discriminatory potential in biomarker development [42].

## DISCUSSION

Modeling suggests that improved tuberculosis diagnostics could reduce morbidity and mortality as dramatically as a novel vaccine or optimized drug regimen [42]. A blood-based diagnostic test could transform care, particularly in children or extrapulmonary disease where sputum production may be difficult or less helpful [2].

Altered glycosylation has been hypothesized to be related to immune activation inducing Abs with unique properties to recruit effector functions [24]. We previously described differences in bulk Ab glycosylation among latent and active tuberculosis [5]. However, it was unclear whether these changes occurred due to alterations in Fab or Fc glycosylation [24] or linked to particular antigen-specific Ab populations [9, 13, 19–22]. Parallel analysis of whole, Fc, and Fab glycans highlighted that the major differences in Ab glycosylation were observed in the Fc, and not the Fab, domain (Figure 1 and Figure 4, and Supplementary Figures 3, 4, and 5). Different Ag specificities (PPD and Ag85A) were associated with divergent IgG glycosylation profiles (Figure 3B and Figure 4). Multivariate modeling using all Ab features further supports that divergent Ab profiles in latent and active tuberculosis, which potentially reflect host immune states, can be identified by analysis of Fc domain glycosylation.

Because the Fab domain is typically highly sialylated and galactosylated compared to the Fc [9, 12, 28], it was unclear if differences in latent and active tuberculosis were associated with increased Fab glycosylation (Figure 1 and Supplementary Figure 1). We found that whole and Fc domain glycans correlated closely with each other (Supplementary Figure 1), more so than whole and Fab domain glycans. Moreover, glycans from whole and Fc but not Fab domains discriminated latent from active tuberculosis (Figure 4B and 4C and Supplementary Figures 3–5). Consistent with these findings, of the 193 Ab features incorporated into the model, 4 of the downselected 5 that were sufficient in discriminating latent and active tuberculosis included Fc or whole but not Fab Ab glycans (Figure 4B). Intriguingly, one feature also selected was Ab-mediated induction of CD107a surface expression on NK cells (Figure 4B), which is associated with activation and consistent with published literature linking NK cell function to latent tuberculosis in humans [5, 43]. Thus, while there is a relative abundance of sialic acid and galactose on Fab domains, Ab glycosylation changes associated with disease resolution occur on the Fc domain and appear to impact Ab functions.

Differences in Ab glycosylation were observed between non-antigen–specific, PPD- and Ag85A-specific IgG glycan profiles (Figure 2 and Supplementary Figure 2). However, the resolving power of antigen-specific whole IgG glycans in this cohort was less than that of non-antigen–specific Fc domain sugar moieties (Figure 4C). This discrepancy may be due to the resolution of whole versus Fc domain antigen-specific glycosylation. While sample quantity in this study was insufficient for this comparison, future work in this direction may provide enhanced resolution. In addition, the nature of the *M. tuberculosis* antigens used may contribute to the lower discriminatory capacity of antigen-specific IgG glycosylation. PPD reflects a breadth of *M. tuberculosis* antigens such that the complexity could obscure differences. In contrast, as 1 of the more than 4000 proteins encoded in the *M. tuberculosis* genome [44], Abs reactive to Ag85A may not capture the extent of variation present against the *M. tuberculosis* antigen repertoire. Furthermore, BCG vaccination, received by 83% of the individuals (Supplementary Table), is likely to have induced humoral responses cross-reactive to both PPD and Ag85A, altering the resolving power of both antigen-specific Ab responses. Additional *M. tuberculosis*-specific targets, such as ESAT6 and CFP10 used in current T-cell based diagnostics, may provide even greater resolution. Future studies with expanded antigen libraries may provide a more concrete path to resolve latent and active tuberculosis in an antigen-specific manner and beyond current transcriptomic and proteomic based biomarker signatures [4, 45–47].

Ab glycosylation, with promise as biomarkers in autoimmunity and cancer [9, 10, 48], represents a diagnostic approach that could complement current management tools for tuberculosis. Given emerging high-throughput chromatographic separation techniques for glycan analysis [28, 49] with the potential to transition into a point-of-care diagnostic [50], Fc glycosylation could be captured to discriminate between latent and active tuberculosis. Notably, *M. tuberculosis* in humans is clinically more than the classic dichotomy of latent infection and active disease. Within this spectrum are individuals who progress, those who potentially regress, and those who never progress. Fc glycans could begin to identify subpopulations with higher risk of progression and thus more benefit from therapy [28]. The best separation was observed when Fc glycosylation was combined with *M. tuberculosis*-specific Ab titers (Figure 4C). These data suggest that both quantitative changes in disease-specific Abs and qualitative changes in the inflammatory state of these Abs provide the greatest resolution, with Fc glycosylation profiling paving a path to point-of-care *M. tuberculosis* diagnostic development.

## Supplementary Data

Supplementary materials are available at *The Journal of Infectious Diseases* online. Consisting of data provided by the authors to benefit the reader, the posted materials are not copyedited and are the sole responsibility of the authors, so questions or comments should be addressed to the corresponding author.

jiz643_suppl_Supplemental_Figure_1

jiz643_suppl_Supplemental_Figure_2

jiz643_suppl_Supplemental_Figure_3

jiz643_suppl_Supplemental_Figure_4

jiz643_suppl_Supplemental_Figure_5

jiz643_suppl_Supplemental_Figure_6

jiz643_suppl_Supplemental_Figure_7

jiz643_suppl_Supplemental_Table

## Notes

***Acknowledgment.*** We thank the health authorities and the staff at the tuberculosis clinics in Matamoros, Tamaulipas, Mexico and Hidalgo county, Texas for their support in participant enrollment.

***Financial support.*** This work was supported by the Gates Foundation (grant numbers OPP1151840 and OPP1156795 to G.A.) at the Ragon Institute; National Institutes of Health (grant number K08-AI130357 to L.L.L.), the Ragon Institute (to G.A. and S.M.F.); and the Samana Cay Massachusetts General Hospital Scholar Program (to G.A.).

***Potential conflicts of interest.*** All authors: No reported conflicts of interest. All authors have submitted the ICMJE Form for Disclosure of Potential Conflicts of Interest. Conflicts that the editors consider relevant to the content of the manuscript have been disclosed.

## References

[1] World Health Organization. Global Tuberculosis Report 2018. Geneva, Switzerland: WHO, 2018.

[2] Lewinsohn DM, Leonard MK, LoBue PA, et al. Official American Thoracic Society/Infectious Diseases Society of America/Centers for Disease Control and Prevention clinical practice guidelines: diagnosis of tuberculosis in adults and children. Clin Infect Dis 2017; 64:111–5. 28052967 10.1093/cid/ciw778 PMC5504475

[3] Tostmann A, Kik SV, Kalisvaart NA, et al. Tuberculosis transmission by patients with smear-negative pulmonary tuberculosis in a large cohort in the Netherlands. Clin Infect Dis 2008; 47:1135–42. 18823268 10.1086/591974

[4] Singhania A, Verma R, Graham CM, et al. A modular transcriptional signature identifies phenotypic heterogeneity of human tuberculosis infection. Nat Commun 2018; 9:2308. 29921861 10.1038/s41467-018-04579-w PMC6008327

[5] Lu LL, Chung AW, Rosebrock TR, et al A functional role for antibodies in tuberculosis. Cell 2016; 167:433–43.e14. 27667685 10.1016/j.cell.2016.08.072 PMC5526202

[6] Walzl G, Haks MC, Joosten SA, Kleynhans L, Ronacher K, Ottenhoff TH Clinical immunology and multiplex biomarkers of human tuberculosis. Cold Spring Harb Perspect Med 2015; 5:pii: a018515. 10.1101/cshperspect.a018515 PMC4382732 25475107

[7] Ernst JD The immunological life cycle of tuberculosis. Nat Rev Immunol 2012; 12:581–91. 22790178 10.1038/nri3259

[8] Broger T, Basu Roy R, Filomena A, et al. Diagnostic performance of tuberculosis-specific IgG antibody profiles in patients with presumptive tuberculosis from two continents. Clin Infect Dis 2017; 64:947–55. 28362937 10.1093/cid/cix023 PMC5848306

[9] Hafkenscheid L, Bondt A, Scherer HU, et al. Structural analysis of variable domain glycosylation of anti-citrullinated protein antibodies in rheumatoid arthritis reveals the presence of highly sialylated glycans. Mol Cell Proteomics 2017; 16:278–87. 27956708 10.1074/mcp.M116.062919 PMC5294214

[10] Wang JR, Gao WN, Grimm R, et al. A method to identify trace sulfated IgG N-glycans as biomarkers for rheumatoid arthritis. Nat Commun 2017; 8:631. 28931878 10.1038/s41467-017-00662-w PMC5606999

[11] Catera M, Borelli V, Malagolini N, et al. Identification of novel plasma glycosylation-associated markers of aging. Oncotarget 2016; 7:7455–68. 26840264 10.18632/oncotarget.7059 PMC4884931

[12] Bondt A, Rombouts Y, Selman MH, et al. Immunoglobulin G (IgG) Fab glycosylation analysis using a new mass spectrometric high-throughput profiling method reveals pregnancy-associated changes. Mol Cell Proteomics 2014; 13:3029–39. 25004930 10.1074/mcp.M114.039537 PMC4223489

[13] Ackerman ME, Crispin M, Yu X, et al. Natural variation in Fc glycosylation of HIV-specific antibodies impacts antiviral activity. J Clin Invest 2013; 123:2183–92. 23563315 10.1172/JCI65708 PMC3637034

[14] Moore JS, Wu X, Kulhavy R, et al. Increased levels of galactose-deficient IgG in sera of HIV-1-infected individuals. AIDS 2005; 19:381–9. 15750391 10.1097/01.aids.0000161767.21405.68

[15] Kumagai T, Palacios A, Casadevall A, et al Serum IgM glycosylation associated with tuberculosis infection in mice. mSphere 2019; 4:pii: e00684-18. 30918063 10.1128/mSphere.00684-18 PMC6437276

[16] Parekh R, Isenberg D, Rook G, Roitt I, Dwek R, Rademacher T A comparative analysis of disease-associated changes in the galactosylation of serum IgG. J Autoimmun 1989; 2:101–14. 10.1016/0896-8411(89)90148-0 2504180

[17] Vidarsson G, Dekkers G, Rispens T IgG subclasses and allotypes: from structure to effector functions. Front Immunol 2014; 5:520. 25368619 10.3389/fimmu.2014.00520 PMC4202688

[18] Pincetic A, Bournazos S, DiLillo DJ, et al. Type I and type II Fc receptors regulate innate and adaptive immunity. Nat Immunol 2014; 15:707–16. 25045879 10.1038/ni.2939 PMC7430760

[19] Selman MH, de Jong SE, Soonawala D, et al. Changes in antigen-specific IgG1 Fc N-glycosylation upon influenza and tetanus vaccination. Mol Cell Proteomics 2012; 11:M111.014563. 10.1074/mcp.M111.014563 PMC3322571 22184099

[20] Mahan AE, Jennewein MF, Suscovich T, et al. Antigen-specific antibody glycosylation is regulated via vaccination. PLoS Pathog 2016; 12:e1005456. 26982805 10.1371/journal.ppat.1005456 PMC4794126

[21] Wang TT, Maamary J, Tan GS, et al. Anti-HA glycoforms drive B cell affinity selection and determine influenza vaccine efficacy. Cell 2015; 162:160–9. 26140596 10.1016/j.cell.2015.06.026 PMC4594835

[22] Wang TT, Sewatanon J, Memoli MJ, et al. IgG antibodies to dengue enhanced for FcγRIIIA binding determine disease severity. Science 2017; 355:395–8. 28126818 10.1126/science.aai8128 PMC5557095

[23] Bondt A, Hafkenscheid L, Falck D, et al. ACPA IgG galactosylation associates with disease activity in pregnant patients with rheumatoid arthritis. Ann Rheum Dis 2018; 77:1130–6. 29615411 10.1136/annrheumdis-2018-212946

[24] van de Bovenkamp FS, Hafkenscheid L, Rispens T, Rombouts Y The emerging importance of IgG Fab glycosylation in immunity. J Immunol 2016; 196:1435–41. 26851295 10.4049/jimmunol.1502136

[25] van de Bovenkamp FS, Derksen NIL, Ooijevaar-de Heer P, et al Adaptive antibody diversification through N-linked glycosylation of the immunoglobulin variable region. Proc Nat Acad Sci USA 2018; 115:1901–6. 29432186 10.1073/pnas.1711720115 PMC5828577

[26] Wiker HG, Harboe M The antigen 85 complex: a major secretion product of *Mycobacterium tuberculosis*. Microbiol Rev 1992; 56:648–61. 1480113 10.1128/mr.56.4.648-661.1992 PMC372892

[27] Restrepo BI, Camerlin AJ, Rahbar MH, et al. Cross-sectional assessment reveals high diabetes prevalence among newly-diagnosed tuberculosis cases. Bull World Health Organ 2011; 89:352–9. 21556303 10.2471/BLT.10.085738 PMC3089389

[28] Mahan AE, Tedesco J, Dionne K, et al. A method for high-throughput, sensitive analysis of IgG Fc and Fab glycosylation by capillary electrophoresis. J Immunol Methods 2015; 417:34–44. 25523925 10.1016/j.jim.2014.12.004 PMC5054724

[29] Brown EP, Licht AF, Dugast AS, et al. High-throughput, multiplexed IgG subclassing of antigen-specific antibodies from clinical samples. J Immunol Methods 2012; 386:117–23. 23023091 10.1016/j.jim.2012.09.007 PMC3475184

[30] Ackerman ME, Moldt B, Wyatt RT, et al. A robust, high-throughput assay to determine the phagocytic activity of clinical antibody samples. J Immunol Methods 2011; 366:8–19. 21192942 10.1016/j.jim.2010.12.016 PMC3050993

[31] Gómez-Román VR, Florese RH, Patterson LJ, et al. A simplified method for the rapid fluorometric assessment of antibody-dependent cell-mediated cytotoxicity. J Immunol Methods 2006; 308:53–67. 16343526 10.1016/j.jim.2005.09.018

[32] Chung AW, Ghebremichael M, Robinson H, et al Polyfunctional Fc-effector profiles mediated by IgG subclass selection distinguish RV144 and VAX003 vaccines. Sci Trans Med 2014; 6:228ra38. 10.1126/scitranslmed.3007736 24648341

[33] Jegaskanda S, Weinfurter JT, Friedrich TC, Kent SJ Antibody-dependent cellular cytotoxicity is associated with control of pandemic H1N1 influenza virus infection of macaques. J Virol 2013; 87:5512–22. 23468501 10.1128/JVI.03030-12 PMC3648138

[34] Tibshirani R Regression shrinkage and selection via the lasso. J R Stat Soc Series B Stat Methodol 1996; 58:267–88.

[35] Cortes C, Vapnik V Support-vector networks. Mach Learn 1995; 20:273–97.

[36] Ackerman ME, Das J, Pittala S, et al. Route of immunization defines multiple mechanisms of vaccine-mediated protection against SIV. Nat Med 2018; 24:1590–8. 30177821 10.1038/s41591-018-0161-0 PMC6482471

[37] Ojala M, Garriga GC Permutation tests for studying classifier performance. J Mach Learn Res 2010; 11:1833–63.

[38] Prasad TS, Verma R, Kumar S, et al. Proteomic analysis of purified protein derivative of *Mycobacterium tuberculosis*. Clin Proteomics 2013; 10:8. 23870090 10.1186/1559-0275-10-8 PMC3729367

[39] Lim JH, Park JK, Jo EK, et al. Purification and immunoreactivity of three components from the 30/32-kilodalton antigen 85 complex in *Mycobacterium tuberculosis*. Infect Immun 1999; 67:6187–90. 10531287 10.1128/iai.67.11.6187-6190.1999 PMC97013

[40] Kimuda SG, Biraro IA, Bagaya BS, Raynes JG, Cose S Characterising antibody avidity in individuals of varied *Mycobacterium tuberculosis* infection status using surface plasmon resonance. PLoS One 2018; 13:e0205102. 30312318 10.1371/journal.pone.0205102 PMC6185725

[41] Fletcher HA, Snowden MA, Landry B, et al T-cell activation is an immune correlate of risk in BCG vaccinated infants. Nat Commun 2016; 7:11290. 27068708 10.1038/ncomms11290 PMC4832066

[42] Lessells RJ, Cooke GS, Newell ML, Godfrey-Faussett P Evaluation of tuberculosis diagnostics: establishing an evidence base around the public health impact. J Infect Dis 2011; 204 (suppl 4):S1187–95. 21996701 10.1093/infdis/jir412 PMC3192544

[43] Roy Chowdhury R, Vallania F, Yang Q, et al. A multi-cohort study of the immune factors associated with *M. tuberculosis* infection outcomes. Nature 2018; 564:E5. 30377311 10.1038/s41586-018-0635-8 PMC6419094

[44] Schubert OT, Mouritsen J, Ludwig C, et al. The Mtb proteome library: a resource of assays to quantify the complete proteome of *Mycobacterium tuberculosis*. Cell Host Microbe 2013; 13:602–12. 23684311 10.1016/j.chom.2013.04.008 PMC3766585

[45] Penn-Nicholson A, Hraha T, Thompson EG, et al; ACS and GC6–74 Cohort Study Groups Discovery and validation of a prognostic proteomic signature for tuberculosis progression: a prospective cohort study. PLoS Med 2019; 16:e1002781. 30990820 10.1371/journal.pmed.1002781 PMC6467365

[46] MacLean E, Broger T, Yerlikaya S, Fernandez-Carballo BL, Pai M, Denkinger CM A systematic review of biomarkers to detect active tuberculosis. Nat Microbiol 2019; 4:748–58. 30804546 10.1038/s41564-019-0380-2

[47] Zak DE, Penn-Nicholson A, Scriba TJ, et al; ACS and GC6-74 Cohort Study Groups A blood RNA signature for tuberculosis disease risk: a prospective cohort study. Lancet 2016; 387:2312–22. 27017310 10.1016/S0140-6736(15)01316-1 PMC5392204

[48] Bones J, Mittermayr S, O’Donoghue N, Guttman A, Rudd PM Ultra performance liquid chromatographic profiling of serum N-glycans for fast and efficient identification of cancer associated alterations in glycosylation. Anal Chem 2010; 82:10208–15. 21073175 10.1021/ac102860w

[49] Reiding KR, Bondt A, Hennig R, et al. High-throughput serum N-glycomics: method comparison and application to study rheumatoid arthritis and pregnancy-associated changes. Mol Cell Proteomics 2019; 18:3–15. 30242110 10.1074/mcp.RA117.000454 PMC6317482

[50] Sarkar A, Lu L, Han J, Alter G An integrated electrical enzyme-linked immunosorbent assay (E2LISA) for point-of-care diagnosis of tuberculosis using glycan biomarkers. 21st International Conference on Miniaturized Systems for Chemistry and Life Sciences (μTAS 2017). Savannah, Georgia, 2017.

