# Supplementary figures and images for: Antibody Fc Glycosylation Discriminates Between Latent and Active Tuberculosis
Source: J Infect Dis. 2020 Feb 15;222(12):2093–102. doi: 10.1093/infdis/jiz643 (PMC7661770; doi:10.1093/infdis/jiz643)

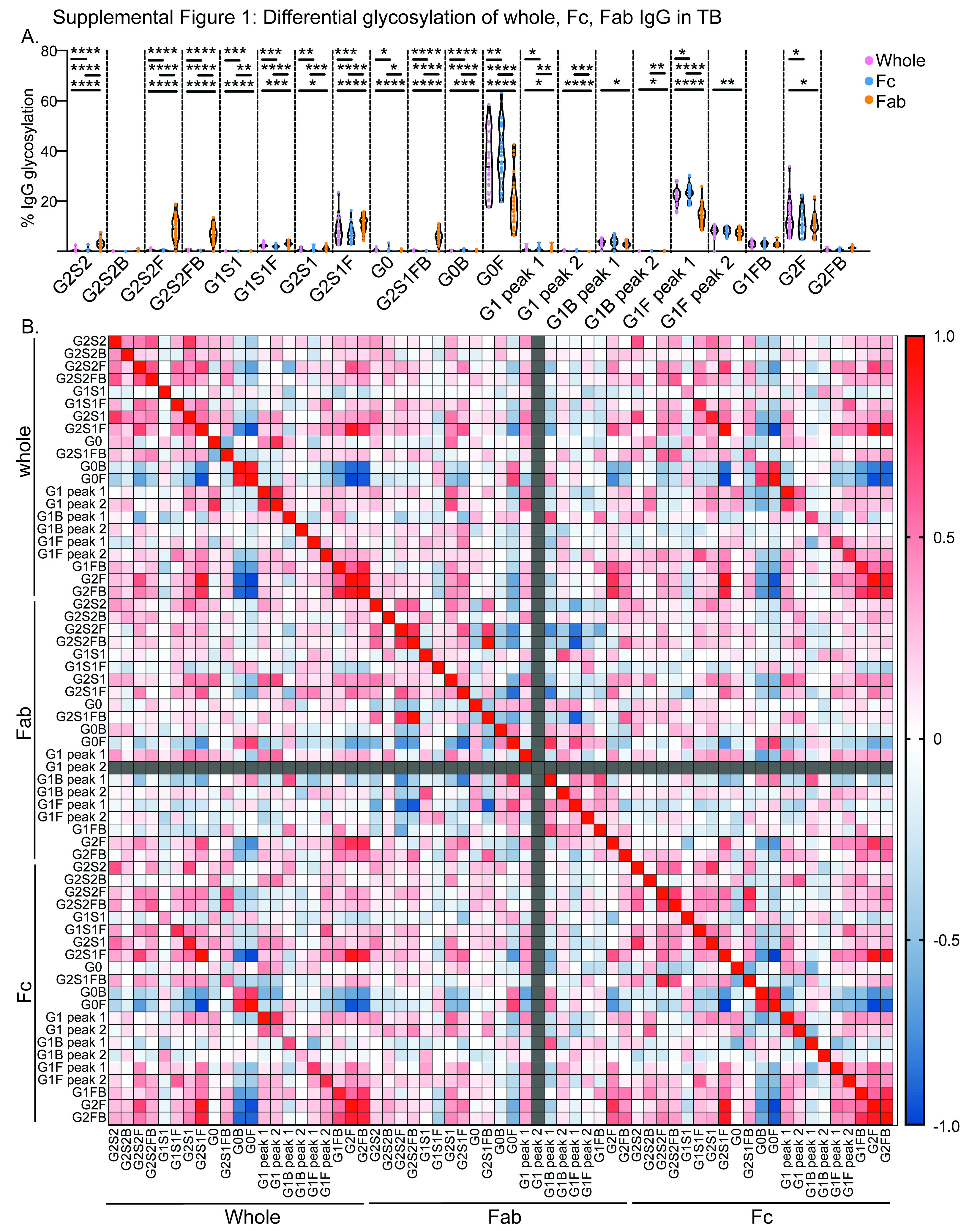

Supplement: jiz643_suppl_Supplemental_Figure_1 [file jiz643_suppl_supplemental_figure_1.jpeg]

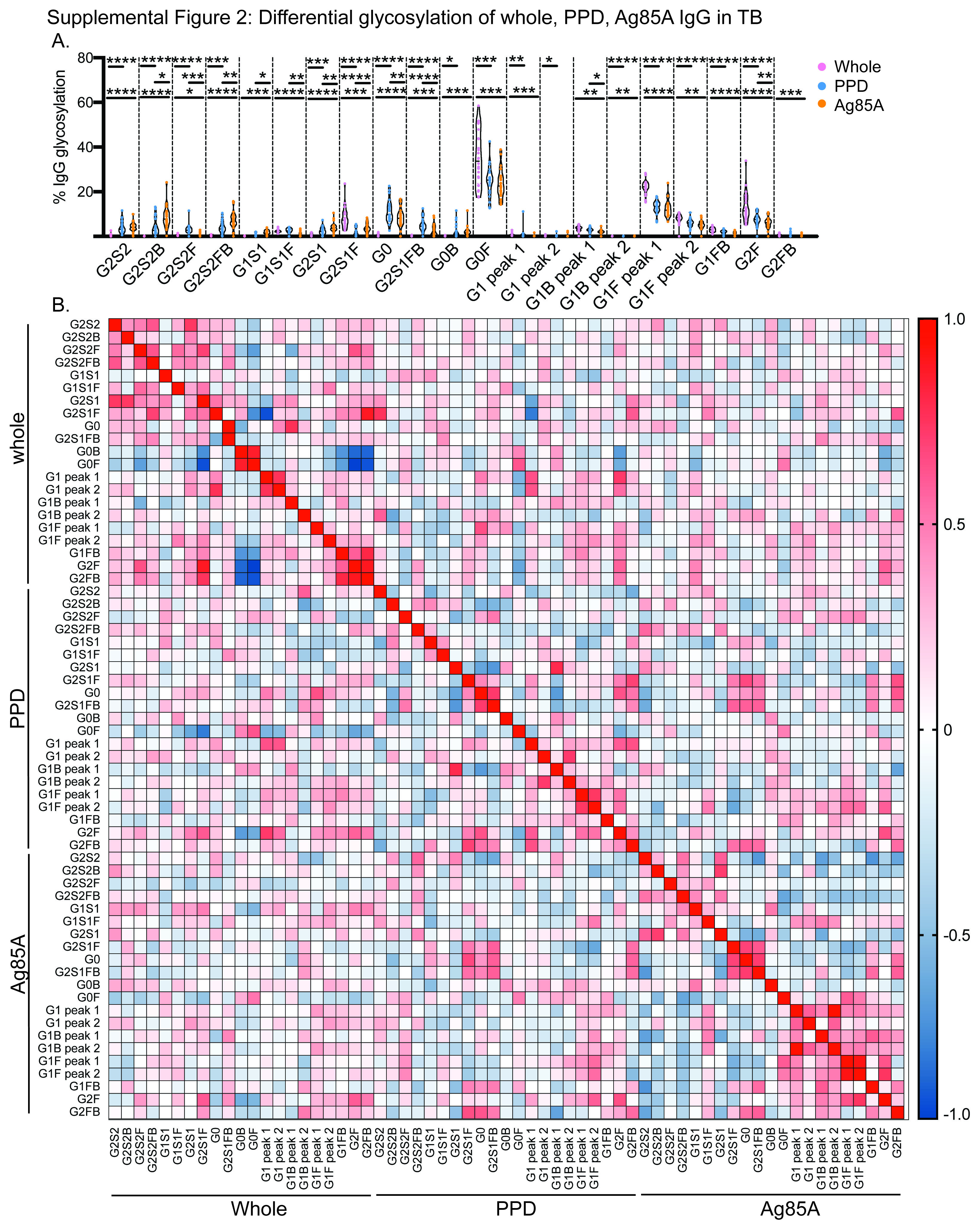

Supplement: jiz643_suppl_Supplemental_Figure_2 [file jiz643_suppl_supplemental_figure_2.jpeg]

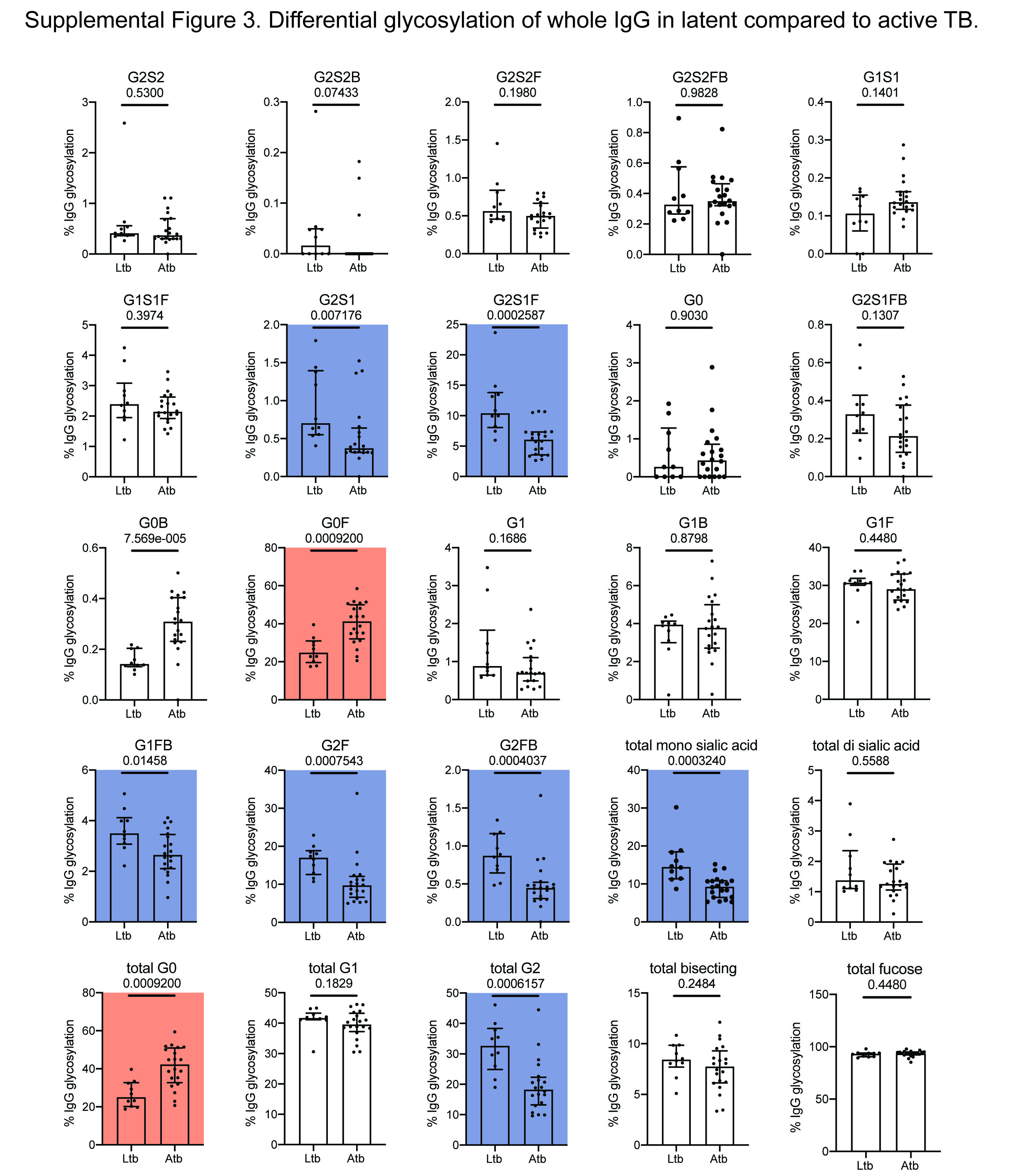

Supplement: jiz643_suppl_Supplemental_Figure_3 [file jiz643_suppl_supplemental_figure_3.jpeg]

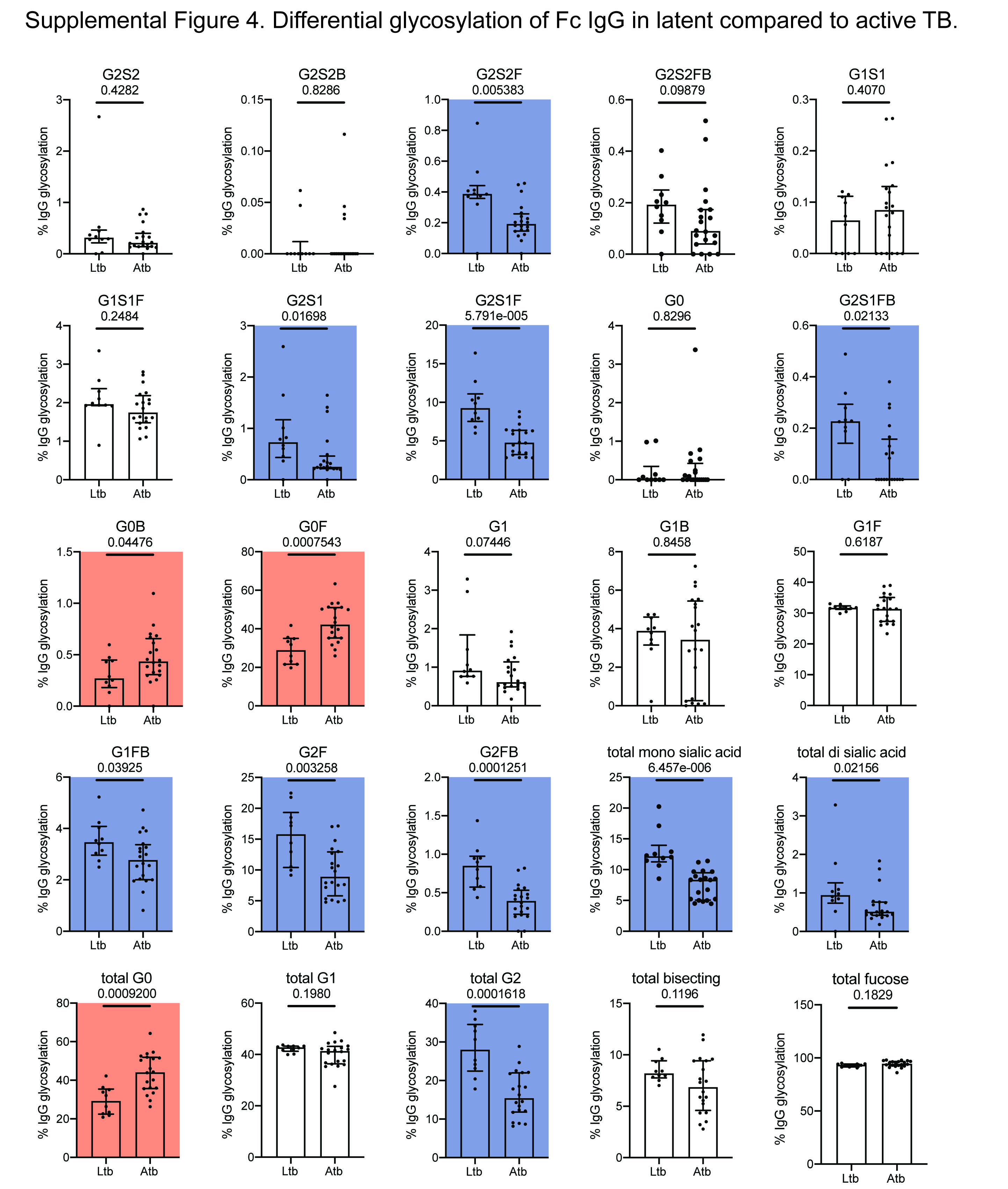

Supplement: jiz643_suppl_Supplemental_Figure_4 [file jiz643_suppl_supplemental_figure_4.jpeg]

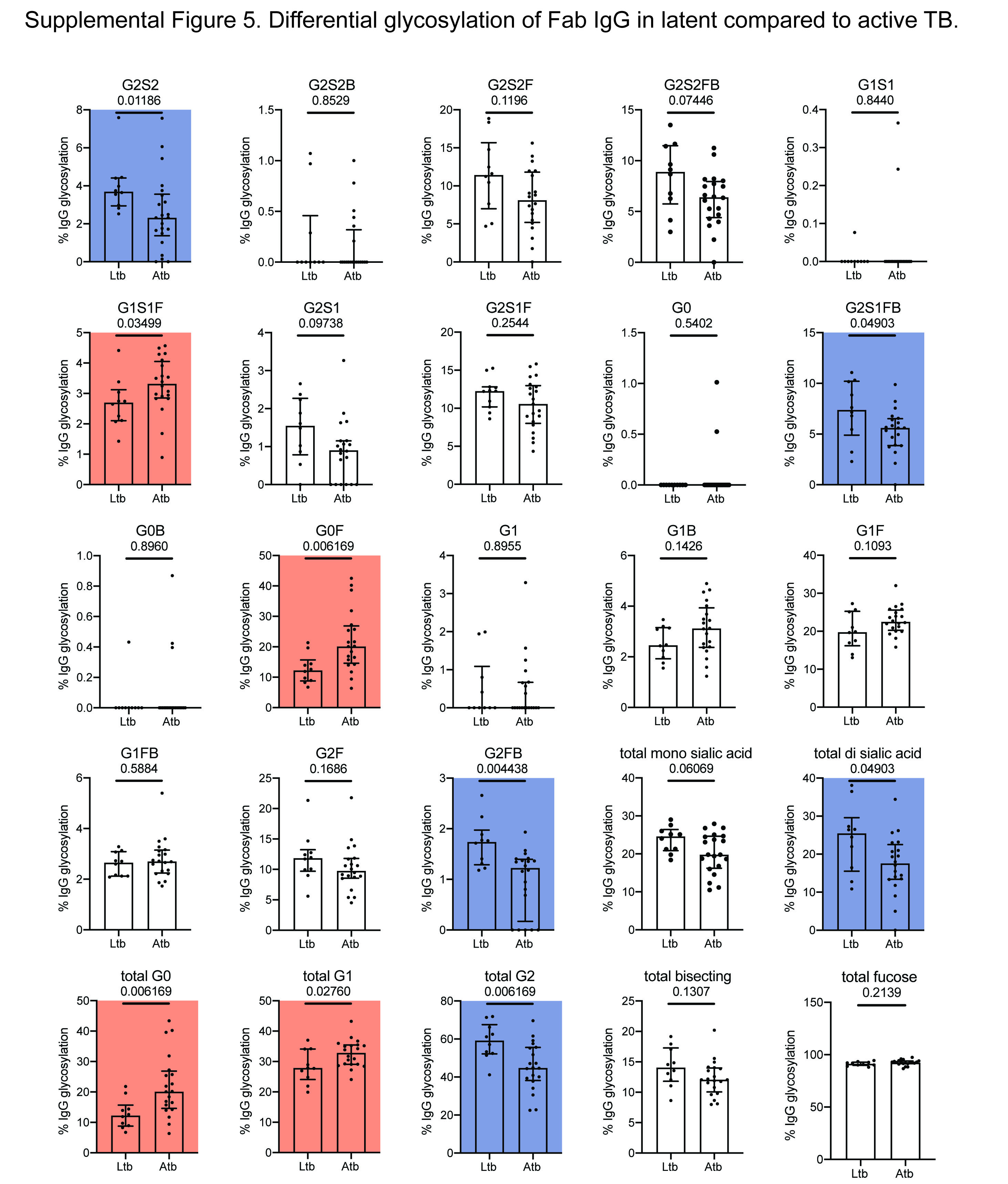

Supplement: jiz643_suppl_Supplemental_Figure_5 [file jiz643_suppl_supplemental_figure_5.jpeg]

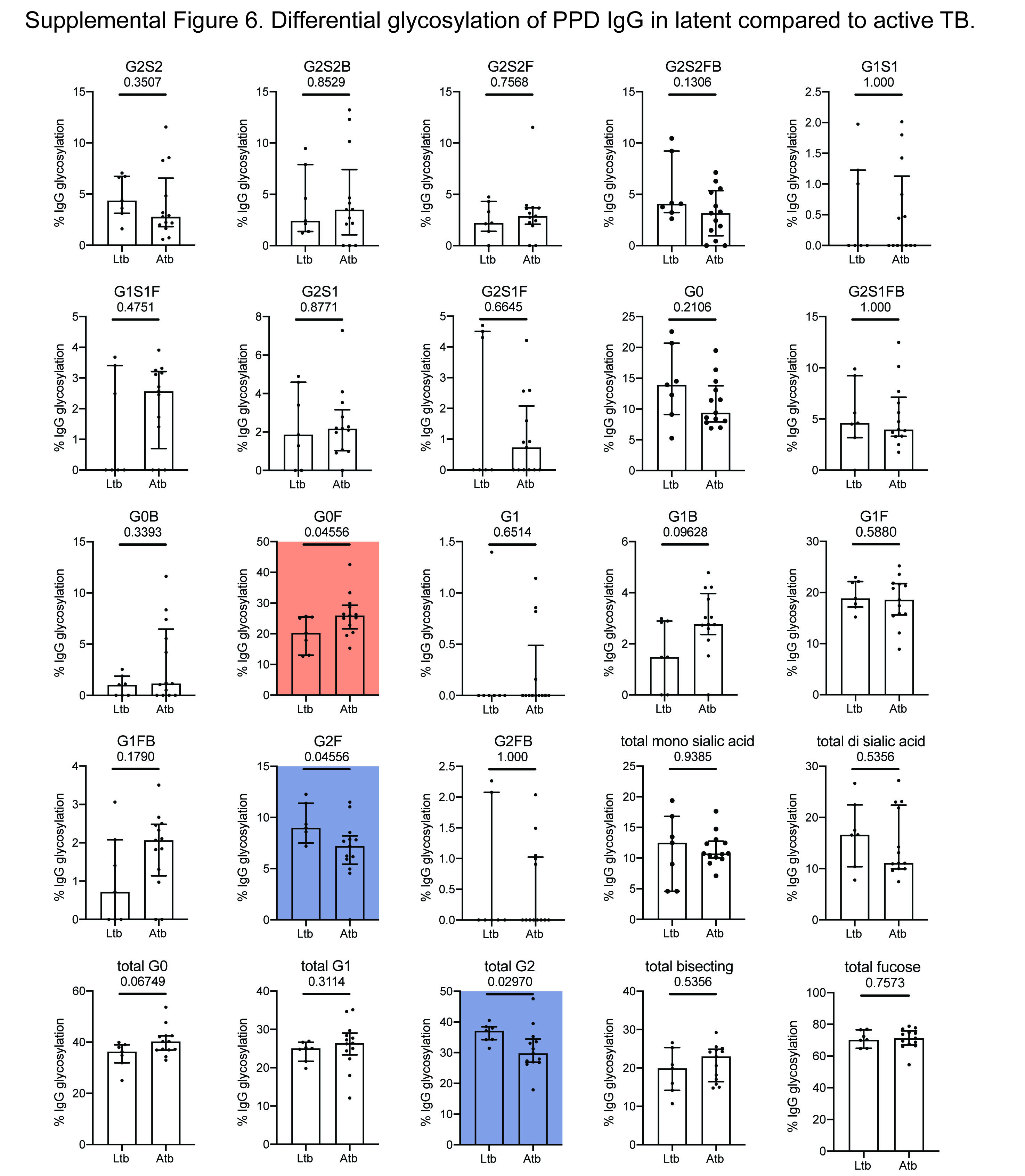

Supplement: jiz643_suppl_Supplemental_Figure_6 [file jiz643_suppl_supplemental_figure_6.jpeg]

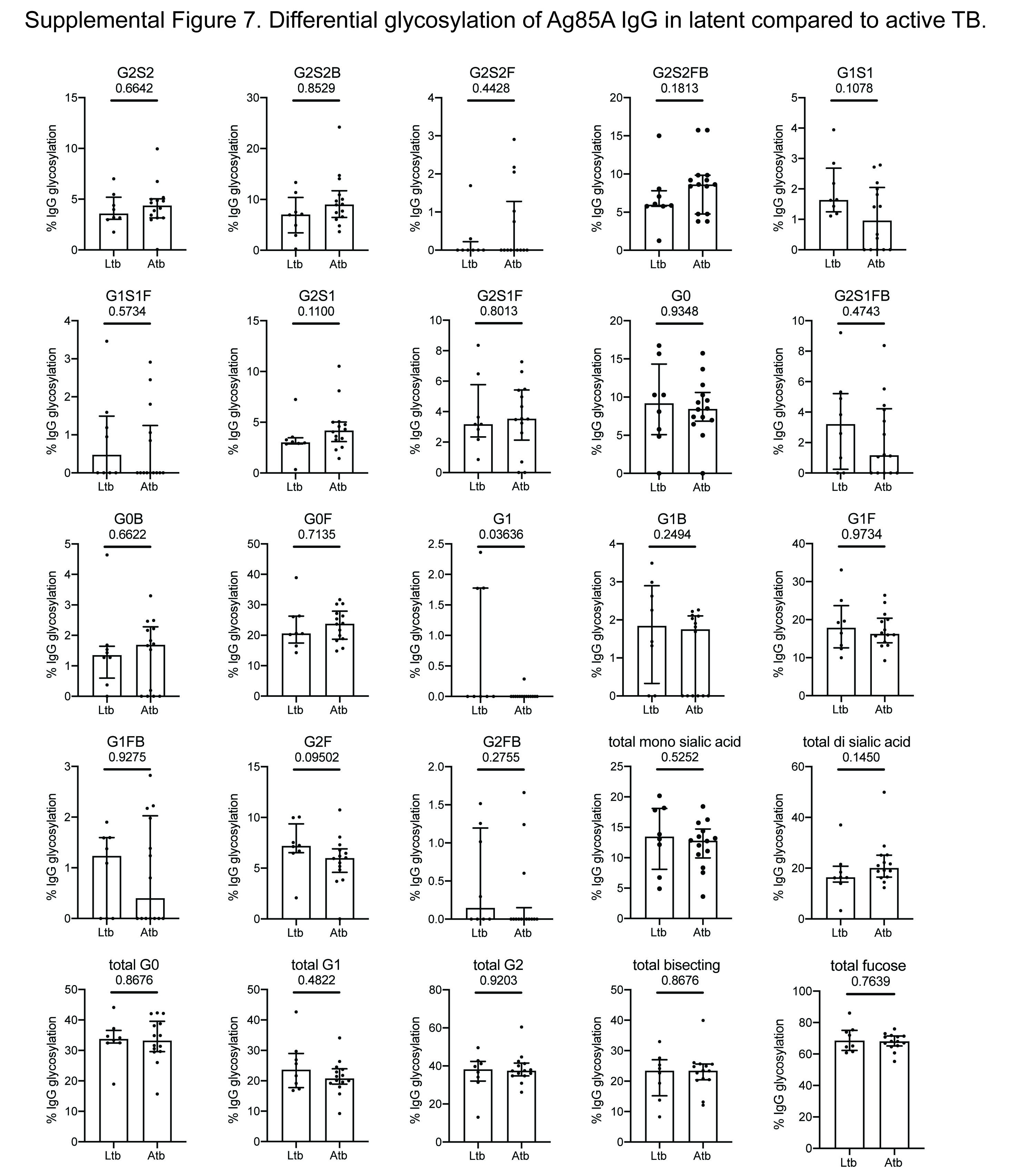

Supplement: jiz643_suppl_Supplemental_Figure_7 [file jiz643_suppl_supplemental_figure_7.jpeg]
